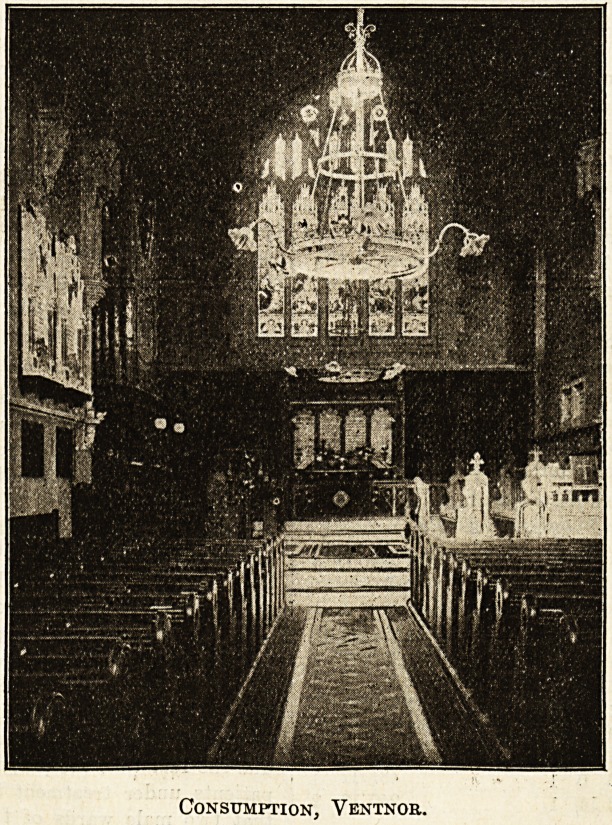# The Royal National Hospital for Consumption, Ventnor

**Published:** 1916-04-08

**Authors:** 


### SOME HOSPITAL CHAPELS.*

### The Royal National Hospital for Consumption, Ventnor.

THE chapel of the Royal National Hospital for Consumption, most appropriately dedicated to St. Luke, occupies a beautiful site within the grounds of the institution in one of the loveliest and most sheltered spots of the Undercliff in the Isle of Wight. It is accessible from every part of the hospital by a covered subway. The building itself, which is furnished with pews and has accommodation for some two hundred persons, is built in the style of the late fourteenth century, with an open roof of timber; it is lighted with electric light, and the organ is placed at the north side of the chancel. A large and beautiful window of five lights, having as its central subject the "Crucifixion," occupies a position at the east end, while numerous other stained-glass windows commemorate deceased benefactors of the hospital. The chapel contains some notable memorial tablets, among them being two which were erected by a member of the Board. The first commemorates the late Queen Victoria, and bears the following inscription: *In Memory of Her Majesty Queen Victoria, Patroness of this Institution for many Years. A Token of Devoted Loyalty and Affection.*

The second was erected in memory of King Edward VII., and bears the Royal Arms, with the following inscription: *In Memory of His Majesty King Edward VII., who gave this Hospital his Gracious Patronage throughout his Reign. A Token of Devoted Loyalty and Affection.*

On the north side is placed a third Royal memorial, oval in design, to the memory of the late Duke of Albany, who was for many years much interested in the work of the institution and, indeed, its second president.

The chapel is fortunate in having recently been greatly enriched by a gift from the late treasurer, Mr. George W. Dawes, J.P., which takes the form of a beautiful Gothic reredos, the chief structure of which is composed of white Carrara marble, and the walls on either side and also on the north and south of the sacrarium have been lined with various coloured marbles, which are thoroughly in keeping with the reredos itself and greatly enhance its appearance. A fine cloister, which has been built by the hospital employees, has also been added, from which the patients are able to obtain a full and uninterrupted view of the hospital grounds, with the English Channel in the distance.

CONSUMPTION, VENTNOR.

The Rev. J. A. Alloway, the chaplain, who was ordained in 1878 to the curacy of Godshill, in the Isle of Wight, in the Diocese of Winchester, which position he held until 1883, when he became chaplain to the Ventnor Hospital, has his room situated as nearly as possible in the middle of the group of buildings, in order that he may be within easy reach of the patients in whichever one of the various houses they may be placed. The chaplain's work at this hospital is never done, for, apart from the actual chapel services, his duties entail continuous and friendly intercourse with the patients, apart from his pastoral visitations, which have a special importance and value.

Every facility is afforded to the inmates of all denominations, when weather and circumstances permit, to attend a place of worship of their own denomination if they so wish, whilst the Jewish patients have special provision made for them.

Another purpose to which the chaplain's room is put is that of a library. This library contains over fifteen hundred volumes, in addition to numerous magazines and periodicals. It is open daily for the use of the patients, who can borrow such volumes as they may select.

* See THE HOSPITAL for May 1, 1915, p. 118.

## Figures and Tables

**Figure f1:**